# Addressing technology-mediated stigma in sexual health-related digital platforms: Insights from design team members

**DOI:** 10.1371/journal.pdig.0000722

**Published:** 2025-02-04

**Authors:** Abdul-Fatawu Abdulai, Amanda Fuchsia Howard, Paul J. Yong, Leanne M. Currie

**Affiliations:** 1 School of Nursing, University of British Columbia, Vancouver, British Columbia, Canada; 2 Department of Obstetrics and Gynaecology, University of British Columbia, Vancouver, British Columbia, Canada; 3 Women Health Research Institute, British Columbia Women’s Hospital & Health Center, Vancouver, British Columbia, Canada; Australian Artificial Intelligence Institute, University of Technology, AUSTRALIA

## Abstract

Digital health technologies are increasingly used as complementary tools in accessing sexual health-related services. At the same time, there are concerns regarding how some interface features and content of these technologies could inadvertently foment stigma among end users. In this study, we explored how design teams (i.e., those involved in creating digital health technologies) might address stigmatizing components when designing sexual health-related digital technologies. We interviewed 14 design team members (i.e., software engineers, user interface and user experience (UI/UX) designers, content creators, and project managers) who were involved in digital health design projects across two universities in western Canada. The interviews sought to undersand their perspectives of how to create destigmatizing digital technologies and were centered on strategies that they might adopt or the kind of expertise or support they might need to be able to address stigmatizing features or content on sexual health-related digital technologies. The findings revealed two overarching approaches regarding how digital health technologies could be designed to prevent the unintended effects of stigma. These include functional design considerations (i.e., pop-up notifications, infographics, and video-based testimonials, and avoiding the use of cookies or other security-risk features) and non-functional design considerations (i.e., adopting an interprofessional and collaborative approach to design, educating software designers on domain knowledge about stigma, and ensuring consistent user testing of content). These findings reflected functional and non-functional design strategies as applied in software design. These findings are considered crucial in addressing stigma but are not often apparent to designers involved in digital health projects. This suggests the need for software engineers to understand and consider non-functional, emotional, and content-related design strategies that could address stigmatizing attributes via digital health platforms.

## Author summary

Digital health technologies are increasingly used for accessing sexual and reproductive health services. At the same time, there are concerns that these technologies can cause and perpetuate stigma among end user patients. In this study, we interviewed 14 digital health development team members to understand their perspectives on how such technologies can be created to prevent the unintended effects of stigma. To prevent technologies from perpetuating stigma, digital health interventions should focus on integrating warning features on digital health platforms, integrating pop-up notifications, patient-centered infographics, video-based testimonials, limiting the use of cookies and enhancing other security-risk features. Other approaches to preventing stigma, particularly during the development process includes interprofessional and collaborative approach to design, educating software designers on domain knowledge about stigma, and ensuring that the content is user tested. These measures, when implemented might help alleviate digital health-facilitated stigma that often emanate from technology development team.

## Introduction

The growth of digital health technologies, such as websites, mobile apps, and social networking sites, has fundamentally changed the way health information is accessed, analyzed, and utilized by patients and healthcare professionals alike [1]. It has been suggested that people are more likely to use technology-based interventions for health problems that are perceived as embarrassing, stigmatizing, and difficult to discuss face-to-face [2,3]. Given the stigma associated with in-person clinical encounters and interpersonal interactions related to sexual health, various digital health interventions have been developed to complement, and in some cases, replace conventional health services [4–6]. Stigma is defined as an attribute and a dynamic social process characterized by widespread social disapproval, blame, rejection, devaluation, and segregation [7]. The extent to which people with sexual health issues are stigmatized depends on the nature of the condition or disorder and whether it is concealed or exposed [7]. People with concealed sexual health-related conditions/disorders are likely to have and/or suffer from internalized stigma while those with exposed conditions are likely to be faced with public stigma (also called enacted stigma) [8]. Public or enacted stigma refers to overt discriminatory practices from the general public that are directed at someone who has a perceived negative condition or disorder, while internalized stigma refers to the implicit acceptance of negative attributes and a reduced sense of self-worth as a result of possessing a supposedly negative attribute [8].

To prevent the stigma associated with visiting conventional health facilities, digital health offers a better alternative to providing stigma-free services at a person’s convenience. At the same time, there are concerns regarding how some digital health platforms or apps could contain interface components that could inadvertently result in stigmatized feelings among end-users [9]. Our previous work shows how some sexual health-related digital health platforms were limited in their ability to address stigma-associated concerns [9]. The inability of digital health platforms to alleviate the stigma of sexual health or the possibility of fomenting stigma suggests a possible lack of awareness about stigma among digital health design team members.

In response to this gap, we developed a set of destigmatizing design guidelines by engaging experts with domain knowledge in stigma and sexual health in 3 rounds of Delphi study [10]. The development of these guidelines was informed by a trauma-informed care framework and they are currently mapped onto Fallot and Harris’s five principles of trauma-informed care framework (i.e., safety, trust, empowerment, collaboration, and choice) [11]. These guidelines are meant to serve as a reference for designing destigmatizing sexual health-related digital platforms that limit the likelihood of fomenting stigma among end-user patients. After obtaining the set of destigmatizing design guidelines, it was important to ascertain how these guidelines can help design team members create sexual health-related digital platforms that reduce the unintended consequences of stigma. Therefore, the purpose of this present study was to answer the research questions—how might these design guidelines guide design teams to create sexual health-related digital platforms that prevent the unintended consequences of stigma? We are focused on digital health design team members because we wanted to understand how those who are responsible for the design, development, and deployment of digital health interventions, appreciate the implications of their design decisions on stigma. The design guidelines from our prior Delphi study are provided in Table 1 below.

**Table 1:** Destigmatizing Design Guidelines by Trauma Informed Care (TIC) Categories.

TIC Category	#	Destigmatizing design guideline
**Emotional safety**	1	Provide participants with the ability for anonymous engagement
	2	Encrypt websites that collect personal information to prevent unauthorized access to personal data math statements
	3	Avoid using language that has a tone of blame or judgment of people living with the condition.
**Choice**	4	Provide contact information for counselors or other psychological supports
	5	Include a range of evidence-based information that touches on different aspects of the condition.
**Trustworthiness**	6	Include information that corrects myths about a condition, to enable users to get accurate information.
	7	Provide factual and plain language information that normalizes and de-stigmatizes sexual health-related conditions.
	8	Selection of language/images should be done in consultation with the community to ensure diversity.
	9	Use images of people from diverse ethnicities, ages, and gender identities who have experienced the condition.
	10	Develop trust by providing options for different gender identities.
	11	Provide trustworthy content by having a reference list for factual information.
	12	Ensure rigorous methods to know the target audience.
	13	Have clear, factual, and neutral information.
**Empowerment**	14	Consider including links to information on the fundamental rights of people affected by or living with the condition.
	15	Use inclusive language that is sensitive to the context of the condition, e.g., partner instead of husband/wife, the person instead of woman/man.
	16	Include videos/testimonials that center on people’s experiences with the condition, including stigma.
	17	Avoid othering and stereotyping people with a sexual health-related condition.
**Collaboration**	18	Interventions for addressing men’s/women’s health or sexual health-related conditions should be developed and delivered in partnership with those living with the condition.
	19	Consider involving all those affected by the condition, not just people with the condition (e.g., partners of people with the condition)

## Materials and methods

We interviewed digital health design team members to understand their perspectives about how the design guidelines could help them to design destigmatizing sexual health-related digital platforms. In this study, digital health development team members include project managers, subject matter experts/content creators, software engineers, and user interface (UI)/user experience (UX) designers who are or were involved in the design of digital health interventions. We targeted people in these different roles because of their unique responsibilities across the various stages of the digital health design lifecycle [12]. Participants were eligible to participate if they completed at least a bachelor’s degree and had at least one year of experience in digital health design, development, or deployment. Recruitment occurred via a purposive and snowballing sampling technique to identify participants with the requisite knowledge and experience in digital health development who would be able to provide information on the phenomenon being investigated. Participants were recruited until we reached data saturation by informational redundancy (i.e., when new data repeat what has been expressed in previous data).

### Ethics statement

This study was approved by the University of British Columbia Behavioral Research Ethics Board (Approval number = H21-02553). All participants provided written consent before participating in this study.

### Data collection

We conducted semi-structured interviews among design team members via Zoom. To provide context for the interview questions, we provided participants with study materials including the destigmatizing design guidelines, a vignette, and a sample website (https://www.optionsforsexualhealth.org/). The vignette and sample website were designed to portray a patient using/interacting with a digital health platform that strives to have a ‘sex positive’ orientation and ultimately seeks to reduce sexual health-related stigma [13]. S1 Table shows the supplementary materials detailing the Vignette for the interviews. Before the interview, we asked participants to review the vignette, the sample website, as well as the design guidelines developed in the Delphi study. Participants were allowed to ask questions on the study materials (particularly the design guidelines) before the interview. We provided these opportunities for clarification because we wanted to make sure that participants had an adequate understanding of the design guidelines to be able to provide informed responses. During the interview, we first asked the participants regarding their general perceptions of the design guidelines. With participants’ awareness of the design guidelines, we asked them to indicate ways, approaches, and strategies in which they could design sexual health-related digital health to address, reduce, or prevent stigma. We also explored the kind of additional expertise, collaborations, or support digital health design teams might need to be able to address stigmatizing features on content sexual health-related digital platforms. Data collection occurred between November – December 2021 and included both audio and video recordings of participants’ interaction with our sample website. The audio recordings were transcribed using TEMI (Temi.org) online transcription software and were exported into NVivo version 12 (Lumivero, LLC). To maintain anonymity, participants’ faces were not captured in the recordings.

### Data analysis

The data were analyzed thematically using Braun & Clarke’s approach to qualitative data analysis [14]. Two authors (AF & LC) each familiarized themselves with the data by reading the transcripts three to four times. Each of us then developed a coding framework inductively from the first four transcripts. The frameworks were discussed before they were applied to the rest of the data to generate initial inductive codes. The codes were later grouped to construct themes through an iterative process. The study team discussed the themes to arrive at a consensus by making sure that the themes were a true reflection of the data. Following the discussions, the themes were then defined and further clustered into main thematic areas depending on their overall importance to the research question. The clustering of the themes moved the categories to thematic areas that represented common participants’ perspectives on how sexual health-related digital technologies could be designed to alleviate or prevent the unintended effects of stigma. To enhance the rigor of the findings, we triangulated the data by watching the video transcripts to support the coding framework, ascertain further information, and clear doubts or discrepancies that surfaced from the thematic analysis. Given that our participants represent people with different expertise in digital health, we also compared and contrasted data across the different participant groups to see similarities and instances where participants’ perspectives differed.

## Results

### Demographic characteristics of participants

A total of 14 participants across two universities in western Canada participated in this study. On average, the interviews lasted 65 minutes per person. The participants included people with expertise in digital health project management, subject matter content writers (clinicians, health researchers), UX/UI designers, and software designers who are or were involved in designing digital health technologies at some point in their careers. All participants were either graduate students or alumni of the two universities. Participants with prior experience in mental health technology design provided useful insights on how digital health platforms could be designed to address stigma, not only for sexual health-related challenges but across other sensitive and potentially stigmatizing health topics. Table 1 shows the demographic characteristics of the participants. To ensure the anonymity of the participants in the reported findings, their names have been replaced with pseudonyms in the narrative.

### Participants’ perceptions regarding the guidelines

There were mixed reactions when participants were asked about their general perceptions of the guidelines. These differences reflected the different areas of specialization of the participants as seen in Table 2. Except for the content writers and project managers, the software designers and UI/UX professionals generally perceived the emotional and content-related design guidelines as beyond their scope of work. At the outset of the interviews, the statement *“This is not within my job description”* was commonly stated among the software designers and the UI/UX designers. The content writer and the project managers on the other hand reiterated the importance of the design guidelines that are meant to educate the team members or produce the right content. Despite the differences in opinions, all the participants acknowledged that the design guidelines were indeed useful and could serve as a reference toolkit for preventing the unintended consequences of stigma when developing digital health platforms for people with highly stigmatized disorders or conditions.

**Table 2:** Demographic Characteristics.

Characteristic	Participants, n = 14
**Continuous Variables**	**Mean (SD)**
Age	29.8 (5.4)
Years of work experience in digital health design	4.30 (1.8)
**Categorical Variables**	**Frequency (%)**
Gender	
Woman	7 (50.0)
Man	6 (42.8)
Non-binary	1 (7.20)
Designation	
Alumni	8 (57.1)
Graduate student	6 (42.9)
Area of specialization in digital health	
Software engineer	4 (28.6)
UI/UX Designer	3 (21.4)
Project Manager	3 (21.4)
Content writers	4 (28.6)
Country of residence	
Canada	12 (85.8)
United Kingdom	1 (7.20)
Swaziland	1 (7.20)

UI = User Interface, UX = User Experience.

### Approaches to address stigma in digital health technologies

Irrespective of participants’ views of the guidelines, they provided various approaches on how an awareness of the guidelines could help them to design destigmatizing sexual health-related digital platforms. These approaches were conceptualized into two main overarching themes that reflect requirement gathering in the software development process. These include functional and non-functional design approaches to addressing stigma [15]. The results are presented in the context of these two main approaches to health information technology design. To provide context, functional requirements are the statements or principles that specify what the system should provide, how it should act in specific situations, and how it should respond to specific commands, while non-functional requirements are the quality constraints and approach the system must meet to promote good user experience [15].

### Theme 1: Functional design approaches to alleviating stigma

Participants indicated functional user-interface design strategies that they thought could help them in designing sexual health-related digital health to prevent the unintended consequences of stigma. These strategies emanated largely from software designers and UI/UX professionals who are often involved in the technical aspects of digital health projects. Two subthemes emerged under the functional design approaches to alleviating stigma.

#### Subtheme 1: Trigger warnings, privacy and security guarantees.

To be able to prevent digital health-mediated stigma, the participants suggested approaches that would prevent digital health users from being suddenly exposed to content that could trigger stigma or from having the feeling that their information would be used to stigmatize them. Some of the most popular design strategies for alleviating stigma via digital platforms were to consider using pop-up notifications on the landing page of digital health platforms and also by discouraging the use of cookies. These strategies reflected design guidelines 2 and 3 as seen in Table 1 above. The participants described that having pop-up messages that appear on digital health platforms with privacy, security, and confidentiality guarantees as well as pop-up messages that show that cookies are not collected can reassure users of the anonymity, safety, and security of sexual health-related digital technologies during their first visits. To emphasize the importance of pop-up notifications, one user interface designer explained how he might design a pop-up notification that would first “*greet website users*” with assurances of emotional safety immediately after they open or log on to the homepage,

“*I would throw a pop-up message to them at the very beginning that ‘you are safe with us, that ‘we won’t be collecting any personal information. This [*the website*] is very protected and encrypted and guarantees your safety.’ This may have an assurance like, okay, this is a reliable source.”*

According to participants, the use of cookies should be discouraged because such features might give users the impression that their data could be collected or accessed by “*people behind the website project*” which could lead to stigmatized feelings. Discouraging the use of cookies was the most commonly expressed design consideration that participants perceived could reduce privacy and security risks that often fuel stigma. A UX professional described the security risk of cookies and how implementation of cookies could be limited to address stigma by stating that,

“*It’s easy to track the data for people. There are many ways like we have cookies and analytics software where we capture all the data. To address stigma, it should not be easy to capture these kinds of information that is personal to people… just capture their engagement, not the personal details.”*

#### Subtheme 2: Positive framing and cultural sensitivity of content.

To be able to address stigma, the participants indicated how an awareness of the guidelines could help them recognize diversity and ensure that web-based content resonates with people with different cultural and sexual identities. To be able to do that, the participants suggested the use of customized infographics and storytelling-based video testimonials that reflect diverse groups. These approaches were made in reference to design guidelines 6, 7, 8, 9, and 16 as outlined in Table 1. According to the participants, customized infographics and storytelling-based video testimonials could alleviate stigma by fostering emotional safety and making people feel engaged and emotionally connected to what other technology users may be recounting. They also attested that customized infographics and storytelling-based video testimonials that are inclusive can offer information that is relevant, and respectful, and allow users to feel seen, understood, and supported in their sexual health journey. One content writer vividly described the essence of customized images and videos by stating that:

“*When a sexual health platform feels not original, when a website feels like these are just photos that are just taken from everywhere, the target audience can feel less trust, can feel disengaged. They might think the website owners are not serious because they’re not putting enough effort into this. So again, custom photos would be my recommendation.”*

While there was a consensus on displaying videos and infographics on the landing pages of digital health platforms, participants varied on the type of videos to include. Participants with prior experience in designing mental health digital platforms were more interested in using customized videos involving real users while those with other backgrounds were inclined to suggest the use of avatars in place of human subjects. This sensitivity to different contexts was evident across the participants who were considering that showing one’s face in a video testimonial might be a source of stigma for the person making the video, so an avatar would be a good substitution if the person with the stigmatizing condition did not want to be featured on a digital health platform, but wanted to convey authentic information.

### Theme 2: Non-functional design considerations to addressing stigma

Participants, particularly project managers, content writers, and UI/UX professionals suggested non-functional approaches through which stigma can be alleviated via the design of digital health. These non-functional approaches were largely made in reference to content-related design guidelines including guidelines 3, 5, 6, 8, 13, 16, and 17 as outlined in Table 1. These non-functional approaches were mainly in the form of support or additional expertise that design team members may need to be able to address stigmatizing features or content on digital health platforms.

#### Sub-theme 1: Identifying stigmatizing content.

The participants indicated that the content of digital health platforms is “*the spot within which stigma could be amplified*”. They therefore suggested approaches including conducting consistent user with end users to identify stigmatizing content. According to the participants, testing out content with end users is the surest way to identify stigmatizing content. Consistent user testing was made in reference to design guidelines 5, 6, 8, 13, 16, and 17 as outlined in Table 1. The participants reiterated that instead of conducting user testing to identify usability and functionality issues, design teams should focus on conducting user testing to identify content that could be stigmatizing to end users. They also suggested that user testing should be done throughout all the stages of the digital health design process rather than limiting it to the end of the design process. One UI designer reiterated the importance of user testing by stating that…

*“…It has to be user-tested as well. You cannot just sit down and write it down and then publish it on the website. You have to put it in front of people, do you think it is coming off as judgemental? Is it accessible language? Um, does it help de-stigmatize sexual health-related conditions? So, I think that’s why there’s a lot of work that goes into this before you are sure you hit the mark. I will move away from limiting user testing to how the website functions to consider including the written content.”*

While there was a consensus on user testing, there was variability about the type of user testing to be adopted for sexual health-related technologies. Some participants, particularly those with prior experience in designing mental health apps, emphasized face-to-face user testing while others were inclined to use analytic tools (i.e., such as UsabilityHub.com or Crazy Egg). It was evident that there was hesitation from some participants to interact face-to-face with people who might have the stigmatizing condition, not because of fear on the part of the participant, but out of deference to the end user who might already be faced with societal or internalized stigma. Even though the participants indicated that analytic tools could enable end users to disclose information that is generally considered stigmatizing, they acknowledged that it might be difficult to identify stigmatizing content by using this method.

#### Sub-theme 2: Collaborative and interprofessional approach to design.

The participants also indicated how an awareness of the design guidelines has brought to the fore the kind of additional expertise they may need to be able to address stigmatizing features or content on digital health platforms. For instance, the participants indicated a need for a collaborative and interprofessional approach to design as one of the appropriate steps to designing destigmatizing digital health platforms. Participants referred to design guidelines 11 and 15 when indicating what additional expertise is needed to address stigma. A UI designer specifically indicated how a content writer can complement their expertise in a way that addresses stigma by stating that….

“*As a designer, the content can be provided to me, and I can just put it up on the website. But since this is about addressing stigma, I think a content writer can re-write the content in a way that is good for the website, with the right language, the right sensitivities of the topics, and at a level everyone can understand. In my other projects, I do everything and that should not be the case. I am a designer, and I may not be able to address stigma but the information we put up should not worsen that either.”*

According to the participants, the design guidelines are so diverse, and interprofessional expertise would be needed to effectively address the stigma that may arise from different aspects of a digital health platform. The need for interprofessional expertise was made evident when the software designers indicated that some of the design guidelines were beyond their job description.

#### Sub-theme 3: Awareness of domain knowledge.

The participants, particularly the software engineers and UI/UX designers reiterated that the design guidelines raised the importance of some education in the domain area of stigma and sexual health. This was particularly made evident by the participants with a limited knowledge of stigma. They asserted that this education would not only help them to be aware of stigmatizing language in sexual health but would also make it easier to recognize and identify possible stigmatizing user interface elements before user testing. The participants particularly welcomed the guidelines that touched on enhancing emotional safety and signaled how those guidelines could help raise their emotional sensitivity to stigma and make them aware of what could end up re-stigmatizing their end. One software design emphasized the importance of education in sexual health by stating that,

*“The education we need is about the subject matter of stigma because this is a new subject matter for us. So, education about the community, the community’s perception of the conditions, education about the diseases themselves, like a medical perspective, sensitivity education, medical knowledge training, what are the facts? What are the truths?”*

*Another participant stated*

*“If you’re working with something as sensitive as sexual health, I think you want to have these ideas in mind so that you are not going off and showing happy, hunky-Dory photos of everybody happy, or you are using very vibrant and super fun colors. Like you are designing a music festival. So, in that way, they* [the guidelines] *heightened my emotional sensitivity to what I’m putting in front of the client. So that’s quite helpful.”*

**Fig 1:**
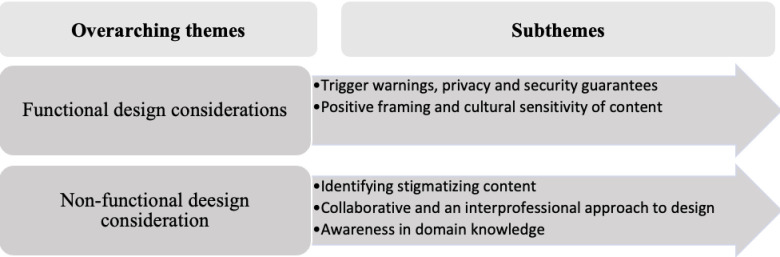
Schematic presentation of the themes.

The findings from this study, which largely reflects functional and non-functional approaches to software design have been depicted in Fig 1 below.

## Discussion

Digital health is frequently used as a complementary and/or alternative means of seeking sexual health services [3,4]. Yet, those involved in the design and development of digital health platforms might not be aware of how their design decisions and the interface content could end up stigmatizing some end users. In this study, we explored how a set of destigmatizing design guidelines developed in a previous study could assist design team members in designing sexual health-related digital health that addresses or prevents the inadvertent effects of stigma. While the participants perceived some of the design guidelines as beyond their scope of practice, they acknowledged that they were a useful reference guide for designing destigmatizing digital health platforms not just for sexual health but for other stigmatizing issues like mental health. The guidelines were considered important because design team members, particularly software designers often consider such guidelines as less obvious, more difficult to specify, and most often ignored by software designers during the design of digital health interventions [15]. While a majority of the guidelines were perceived as a useful reference guide, design guidelines including guidelines 4, 10, 12, 14, 18, and 19 were seen as difficult and not helpful enough in designing destigmatizing design guidelines.

Overall, the findings reflected two main thematic areas as often applied in software design—functional and non-functional design [15,16]. The thematic areas of functional and non-functional design strategies reflected the different roles that the participant groups play in digital health design and development. The software designers and UI/UX professionals were more articulate about technical/functional strategies that can be adopted to address stigma while the project managers and context writers emphasized how content-related/emotional aspects of the principles can help address stigma. The varied opinions of the participants also reinforce the complementary roles of different stakeholders with varied expertise in developing digital health interventions [17,18]. The strategies suggested by the participants show the importance design team members place on addressing digital technology-mediated stigma, which is becoming common but not often recognized in digital health interventions’ development. Other than just emphasizing on efficiency and functionality of digital health platforms, design team members would now see the importance of other non-functional guidelines in addressing abstract issues like stigma. The approaches that design team members can adopt to design destigmatizing digital health platforms, which mirrors the functional and non-functional approaches in software development [15,16], are discussed below.

### Functional approaches to alleviating stigma via digital health platforms

The functional approaches to design emanated largely from participants who were software designers and UI/UX designers. These strategies reflected some of the approaches that software engineers commonly carry out in software engineering [16,19,20]. In the healthcare context, functional design strategies like pop-up notifications have been successfully applied in digital health platforms including electronic medical records and clinical decision support systems [21–23]. The success of these strategies in preventing unintended consequences in other digital technologies suggests that they could be adapted to warn technology users about possible content that could be stigmatizing. It is important to emphasize that integrating cautionary trigger warnings, controlling or preventing the use of cookies, and displaying patient-centered infographics and videos on landing pages of healthcare platforms could foster emotional safety and prevent stigmatized feelings for people who visit sexual health-related digital health platforms [24].

While people with sexual health-related conditions may overtly experience stigma from the public [25], the findings from this study were largely focused on addressing the internalized stigma people might experience after visiting a digital health platform. Internalized stigma refers to the shame, depersonalization, reduction of self-worth, and psychological distress associated with being exposed to something that is perceived as embarrassing [26,27]. Indeed, internalized stigma represents the most common and most difficult form of stigma to be addressed in the context of sexual health [27–29]. It can be argued that most functional design strategies suggested by the participants like pop-up notifications are focused on addressing internalized stigma that may arise from viewing undesirable content. For instance, having trigger warnings that pop up upon visiting a digital health platform would assure users of the security and confidentiality of digital health platforms—reducing the likelihood of being stigmatized from exposure to unpleasant content. Addressing internalized stigma is essential because it is considered the most common and often the most difficult form of stigma to be addressed in the context of sexual health [30].

One important consideration in addressing stigma on digital platforms was positive framing and cultural sensitivity of content. Specific strategies including infographics and video testimonials were emphasized by participants. Participants’ reference to these design strategies reflects inclusive design guidelines often adopted in digital health design [5,31]. Despite the importance of custom-based infographics and video testimonials, there is the risk that such considerations could inadvertently foment or aggravate stigma if they contain images or videos that don’t “sit well” with end users or end up stigmatizing a particular population group [32]. Therefore, the question isn’t about whether to consider custom-based infographics and video testimonials but the kind of infographics to display that would not re-traumatize end users. This point is crucial because there is often a challenge in obtaining real images and videos from individuals who may experience stigma [33]. Thus, further research is warranted to investigate alternate and possibly creative ways of making custom-based video testimonials, perhaps through animation, metaphorical images, or the use of actors, to portray sensitive and personal experiences.

### Non-functional approaches to addressing stigma via digital platforms

The non-functional design approaches, including identifying stigmatizing content via consistent user testing, collaborative approach to design, and education of software engineers were all deemed as important in preventing the unintended effect of stigma via digital health. We consider these as non-functional because they guide how a digital health platform could be designed and do not specify the behavior of the system [19]. Indeed, user testing is quite common in sexual health-related digital health platforms but such activities are often limited to identifying usability and functionality problems and rarely assess whether the content could be stigmatizing to end users [34,35]. This study extended the focus of user testing from identifying usability issues to include testing for content in all stages of the design process. This is important because the best way to identify stigmatizing content is to test such content with the potential end users to determine which aspects come up as stigmatizing. Consistent user testing reiterated the importance of user-driven approaches to designing digital health platforms on potentially stigmatizing topics in sexual health. This process is also being discussed in the literature about radical participatory design, where the design process is partially co-lead by the community and partially co-lead by the designers [36]. While consistent user testing might help identify stigmatizing content, we acknowledge that this might be difficult to carry out in practice because testing all content areas of digital health could be cost- and labor-intensive and could prolong the software development lifecycle. This may be a particular concern in financially driven digital health projects that tend to come with strict timelines and budget constraints [37].

Due to software engineers’ limited understanding of stigma and sexual health, an interprofessional approach was also deemed necessary if stigma alleviation is to be achieved. Indeed, the participants viewed the design guidelines as an educational document that seeks to raise their awareness on sexual health and stigma. The need for education in the domain area of stigma and sexual health also reflects the universal call for ethics education, empathy, and awareness of privacy policies among software engineers involved in developing healthcare technologies [23,24,38]. Even if such education does not directly lead to de-stigmatizing digital health platforms, at least it could support design team members to assess the goodness and consequences of their design decisions on stigma [39]. Indeed, ethical decision-making and sensitivity to stigmatizing content on digital health platforms are considered necessary precursors to a good healthcare system [40]. Additionally, interprofessional collaboration was also deemed as important in addressing technology-mediated stigma. It is worthwhile to state that inter-professional collaboration may necessarily be a new concept in digital health projects [41]. However, such approaches are rarely focused on identifying or addressing complex and abstract technology-related problems like stigma. With this study, technology developers can now have guiding strategies on how to address other abstract patient concerns like stigma. Our team’s recent work on how to address abstract patient concerns like trauma shows that addressing stigma is possible with an interprofessional approach [42]. For instance, in an interprofessional design team, a content writer can re-write content and structure the interface layout in a non-stigmatizing language while a software designer encodes the content into the user interface [43].

## Limitations

This study has some limitations that should be taken into consideration when interpreting the findings. While our study materials (i.e., destigmatizing design guidelines, vignette, and sample website) provided a context for the study, we believe that these materials might have biased participants’ responses towards the website used in this study. For example, in reading the destigmatizing design guidelines, the participants may have interpreted the positionality of the researchers and worked to agree with the researchers which might have invoked social desirability bias. Another limitation of this study is the general definition of stigma and sexual health upon which the study was based. Given that stigma is context-specific [28,44,45], some of the destigmatizing design approaches recommended by participants might not apply to some disease-specific digital health platforms developed in settings that are different from the study context. While this was noted as a limitation, we thought a broader focus was needed as a starting point for future work in addressing technology-related stigma for specific sexual health-related conditions/disorders.

## Conclusion

In this study, we explored how sexual health-related digital platforms could be designed to address or prevent the inadvertent effects of stigma. Participants recommended several approaches and the support needed in designing destigmatizing sexual health-related digital technologies. These include adopting an interprofessional and collaborative approach to design, educating software designers on domain knowledge, consistent user testing of content, and adopting technical design strategies like pop-up notifications, using infographics and video-based testimonials, and avoiding the use of cookies or other security-risk features. These strategies will not only help in designing destigmatizing sexual health-related platforms but might also raise the awareness of technical design team members to possible elements that could end up fomenting stigma among end-users. These findings provide insight for designing new or adapting existing sexual health-related platforms.

## Supplementary material

S1 Table Vignette for Interviews. (DOCX)
