# Supplementary material for: Addressing technology-mediated stigma in sexual health-related digital platforms: Insights from design team members
Source: PLOS Digit Health. 2025 Feb 4;4(2):e0000722. doi: 10.1371/journal.pdig.0000722 (PMC11793748; doi:10.1371/journal.pdig.0000722)
Supplement: S1 Table — (DOCX) [file pdig.0000722.s001.docx]

**Vignette for Interviews**

Sam is living with HIV/AIDS and does not visit conventional health facilities for fear of stigma. The options for sexual health (<https://www.optionsforsexualhealth.org/>) were developed to provide evidence-based information and clinical services for people with sexual and reproductive health needs. Sam is now a regular visitor of the website but she still experiences sexual health-related stigma. When questioned further, she does not know what bothers her but the website, in general, bothers her.

As a design team member, you have been tasked to redesign this website in a manner that addresses stigma. You have been presented with a set of 19 destigmatizing design guidelines that will serve as a reference guide in your redesign of the website. Using this website as an example, we are interested in the approaches you will adopt or the expertise/support you might need to be able to address stigmatizing attributes in online platforms related to sexual health.
